# Exploring Cholinergic Compounds for Peripheral Neuropathic Pain Management: A Comprehensive Scoping Review of Rodent Model Studies

**DOI:** 10.3390/ph16101363

**Published:** 2023-09-27

**Authors:** Edouard Montigné, David Balayssac

**Affiliations:** 1 INSERM, U1107, NEURO-DOL, Université Clermont Auvergne, F-63000 Clermont-Ferrand, France; edouard.montigne@gmail.com; 2 INSERM, U1107, NEURO-DOL, Université Clermont Auvergne, Direction de la Recherche Clinique et de l’Innovation, CHU Clermont-Ferrand, F-63000 Clermont-Ferrand, France

**Keywords:** acetylcholine, muscarinic acetylcholine receptor, nicotinic acetylcholine receptor, peripheral neuropathic pain, rodents

## Abstract

Neuropathic pain affects about 7–8% of the population, and its management still poses challenges with unmet needs. Over the past decades, researchers have explored the cholinergic system (muscarinic and nicotinic acetylcholine receptors: mAChR and nAChR) and compounds targeting these receptors as potential analgesics for neuropathic pain management. This scoping review aims to provide an overview of studies on peripheral neuropathic pain (PNP) in rodent models, exploring compounds targeting cholinergic neurotransmission. The inclusion criteria were original articles on PNP in rodent models that explored the use of compounds directly targeting cholinergic neurotransmission and reported results of nociceptive behavioral assays. The literature search was performed in the PubMed and Web of Science databases (1 January 2000–22 April 2023). The selection process yielded 82 publications, encompassing 62 compounds. The most studied compounds were agonists of α4β2 nAChR and α7 nAChR, and antagonists of α9/α10 nAChR, along with those increasing acetylcholine and targeting mAChRs. Studies mainly reported antinociceptive effects in traumatic PNP models, and to a lesser extent, chemotherapy-induced neuropathy or diabetic models. These preclinical studies underscore the considerable potential of cholinergic compounds in the management of PNP, warranting the initiation of clinical trials.

## 1. Introduction

The International Association for the Study of Pain (IASP) defines pain as “an unpleasant sensory and emotional experience associated with, or resembling that associated with, actual or potential tissue damage”. In particular, peripheral neuropathic pain (PNP) is defined as “a direct consequence of a lesion or disease of the somatosensory nervous system and may be felt in areas with no tissue damage”, and more precisely, in the peripheral nervous system (PNS) [1]. These injuries of the peripheral somatosensory system result in maladaptive responses that can cause pain that is either spontaneous or evoked by sensory stimuli, as an increased response to a painful stimulus (hyperalgesia), or a painful response to a normally nonpainful stimulus (allodynia) [2].

Many etiologies can explain PNP, among which traumatic causes (e.g., phantom limb pain, postsurgical/traumatic neuropathy), toxic causes (e.g., chemotherapy-induced peripheral neuropathy—CIPN), and metabolic causes (e.g., hyperglycemic conditions) are found [3]. 

Approximately 7–8% of the general population worldwide experience PNP, with prevalence rates ranging from 0.9% to 17.9% [4]. Guidelines for the first-line pharmacological management of PNP include gabapentin, gabapentin extended release/enacarbil, pregabalin, serotonin and norepinephrine reuptake inhibitors (duloxetine, venlafaxine), and tricyclic antidepressants [5]. However, the efficacy of therapeutic strategies remains limited, with several and underassessed safety concerns [6,7,8], and innovative strategies are particularly needed [9].

The involvement of cholinergic neurotransmission in pain processes has been shown in many studies and could become a new therapeutic target for the management of PNP (for a review, see [10]).

Acetylcholine acts as one of the most prominent neurotransmitters in both the central nervous system (CNS) and PNS, particularly at the ganglionic level of the autonomous nervous system. After its release from cholinergic cells, acetylcholine is quickly metabolized by acetylcholine esterase (AChE) into choline and acetate, and thereafter, biosynthesized to acetylcholine by choline acetyltransferase (ChAT) at the neuronal level. Cholinergic neurotransmission involves two types of receptors: nicotinic acetylcholine receptors (nAChRs), and muscarinic acetylcholine receptors (mAChRs) [11].

The nAChRs are ionotropic receptors consisting of five individual subunits that arrange in a homo- or heteromeric combination and belong to the superfamily of pentameric ligand-gated ion channels (pLGICs), also known in vertebrates as the Cys-loop family. The member subunits of the Cys-loop family contain a disulfide cysteine bridge in the extracellular domain which closes a loop comprising 13 amino acids. Besides the nAChRs, the Cys-loop family includes the serotonin type 3 receptors (5-HT3), gamma aminobutyric acid type A receptors (GABA_A_) and glycine receptors [12]. A total of 19 different nAChR subunits have been identified: α1–α10, β1–β4, γ, δ, and ε subunits [12]. Myriad nAChR subtypes can be formed by different combinations of these subunits. For example, α4 and β2 subunits assemble together and comprise the most abundant nAChR subtype, α4β2 [13]. nAChRs regulate the flow of mainly sodium, potassium and calcium ions across the cell membrane [14].

The mAChRs are metabotropic receptors and are represented by five types of receptors, including m1AChR, m2AChR, m3AChR, m4AChR, and m5AChR. mAChRs are separated into two subtypes: excitatory (m1AChR, m3AChR, and m5AChR), and inhibitory (m2AChR and m4AChR). m1AChR, m3AChR, and m5AChR couple to a Gq protein, activating the phospholipase C pathway and increasing the intracellular calcium concentration. The m2AChRs and m4AChRs receptors couple to a Gi/o protein, inhibiting the adenylate cyclase pathway and resulting in a decrease in the amount of intracellular cyclic adenosine monophosphate [11,15].

The implications of the acetylcholine neurotransmission in pain have been demonstrated with a tonic cholinergic inhibition of spinal nociceptive transmission in rats [16], and nerve injury-induced loss of this cholinergic tone underlies the analgesic effects of cholinergic agonists in neuropathic pain [17,18]. Muscarinic signaling is vital in pain modulation, with direct activation of mAChRs reducing pain and mAChRs inhibition inducing nociceptive hypersensitivity [19]. The antinociceptive effects of systemically administered donepezil (AChE inhibitor) are linked to GABAergic signaling downstream of mAChRs, suggesting that cholinergic neurons’ inhibitory action may be mediated through GABA release [20]. Intrathecally administered AChE inhibitors, like neostigmine, reduce inflammatory hypersensitivity primarily through m2AChR activation in the spinal cord [21]. The m2AChR and m4AChR receptors predominantly mediate antinociception, while m1AChR and m3AChR have minimal involvement in this process [22].

At the spinal level, presynaptic cholinergic receptors play a significant role in modulating nociceptive transmission, with reports of both excitation and inhibition. Neuronal subpopulations in the dorsal root ganglia (DRG) express α7 nAChRs and respond to α7-selective positive allosteric modulators with a calcium rise, but the functional significance remains unclear [23]. mAChRs in the DRG and trigeminal ganglia modulate primary afferent input onto spinal or medullary dorsal horn neurons, leading to inhibition of glutamate release [24,25]. Activation of m2AChR and m4AchR inhibits excitatory synaptic inputs from primary afferents into the spinal cord by targeting voltage-gated calcium channels in primary sensory neurons [26]. Additionally, the m5AchR subtype has a more complex role, mediating positive effects on primary afferent terminals while potentially increasing glutamate release from spinal interneurons [26].

Supraspinal centers, such as the serotonergic raphe nuclei and adrenergic centers in the locus coeruleus and rostral ventromedial medulla (RVM), play a key role in endogenous nociception control through descending modulation of spinal function. Nicotinic cholinergic signaling in brainstem nuclei stimulates descending inhibitory pathways and mediates antinociceptive effects via interactions with α2 adrenergic, 5-HT1c/2, and 5-HT3 serotonergic receptors, and m2AchR in the lumbar spinal cord [27]. Studies also show antinociceptive effects from nAChR agonists in the RVM via activation of α4β2 nAChRs (and to a lesser extent, via α7 nAChRs) [28]. Finally, ChAT-Cre mice have demonstrated direct descending control of spinal sensory transmission by brainstem cholinergic neurons [29].

This scoping review aims to provide an overview of studies conducted in rodent PNP models exploring pharmacological compounds that target cholinergic neurotransmission, including the modulation of acetylcholine neurotransmission, nAChRs, and mAChRs.

## 2. Materials and Methods

We followed the Preferred Reporting Items for Systematic Review and Meta-Analysis extension for Scoping Reviews (PRISMA-ScR) guidelines in the study process [30].

### 2.1. Eligibility Criteria

The inclusion criteria encompassed original articles on PNP in rodent models (in vivo) that investigated the use of compounds directly targeting cholinergic neurotransmission and reported results of nociceptive behavioral assays.

The exclusion criteria encompassed non-English manuscripts, specific types of manuscripts (reviews, meta-analyses, case reports, books, and conference abstracts), publications lacking available abstracts, in vitro or ex vivo studies, and medicinal chemistry reports. Additionally, publications indirectly exploring cholinergic neurotransmission were excluded from the systematic review. For instance, studies using AChRs antagonists to investigate the involvement of AChRs in the analgesic effect of non-cholinergic compounds were not included.

### 2.2. Information Sources and Search Strategy

We conducted literature retrieval in the following databases: PubMed (MEDLINE, National Library of Medicine), and Web of Science (Clarivate Analytics PLC). No registers, websites, organizations, reference lists, or other sources were used. The bibliographical search was performed on 22 April 2023, and starting from 1 January 2000.

For PubMed, the sequence of keywords was “(acetylcholine) and ((neuropathy) or (neuropathic pain)) and ((rat) or (mouse)) and ((muscarinic) or (nicotinic))”. For Web of Science, the sequence of keywords was “*acetylcholine (All Fields) AND (neuropathic pain) or (neuropathy) (All Fields) AND (rat) or (mouse) (All Fields) AND (muscarinic) or (nicotinic) (All Fields)*”.

### 2.3. Study Selection, Data Collection Process and Data Items

All the references from PubMed and Web of Science were extracted and organized using Zotero software (version 6.0.26, Roy Rosenzweig Center for History and New Media) to create a Zotero bibliographic database. This database included the following details for each publication: authors, title, journal, year, abstract, and DOI (Digital Object Identifier). Subsequently, this Zotero bibliographic database was exported to Excel software (version 2021, Microsoft) for analysis.

Initial publication selection, based on title and abstract, was carried out by the authors EM and DB. Following this initial screening, all the authors conducted a second round of selection based on the full-text of the publications and in accordance with the inclusion/exclusion criteria. In cases where discrepancies regarding the inclusion/exclusion criteria arose for a publication, a consensus among the authors was sought to determine whether to include or exclude the publication.

The researchers extracted the following data from the included manuscripts: title, author, year, DOI, type of PNP, rodent species, sex of animals, name of the compounds, targeted AChRs, behavioral tests and main results.

## 3. Results

### 3.1. Study Selection and Characteristics

**Figure 1:**
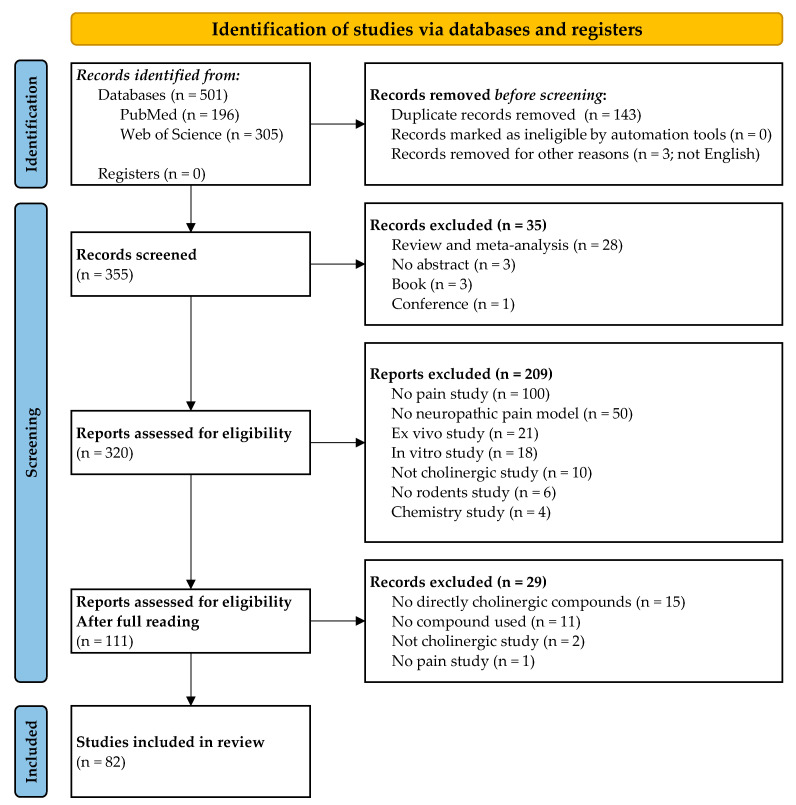
PRISMA flow chart of study selection.

The process for selection and inclusion of publications is presented in Figure 1. A total of 82 publications were selected and analyzed, and these encompassed 62 cholinergic compounds.

Among the publications selected, 58 used animal models of traumatic PNP, 23 CIPN models and 5 diabetic PNP models (Supplementary Materials, Table S1).

For traumatic PNP, nine different models were used as follows:

- Chronic constriction injury (CCI, *n* = 24);
- Partial sciatic nerve ligation (PSL, *n* = 18);
- Spinal nerve ligation (SNL, *n* = 13);
- Spared nerve injury (SNI, *n* = 2);
- Common peroneal nerve ligation (CPNL, *n* = 2);
- Cuff model (CM, *n* = 1);
- Sciatic nerve crush injury (SCNI, *n* = 1);
- Sciatic nerve transection (SNT, *n* = 1);
- Tibial nerve transection (TNT, *n* = 1).

Among the CIPN models, the oxaliplatin model (*n* = 14) was the most used, but other models such as paclitaxel (*n* = 6), vincristine (*n* = 4), and bortezomib (*n* = 1) were also described. Finally, two models of diabetic PNP were used: streptozotocin-induced peripheral neuropathy (SZT, *n* = 4), and the high-fat diet (HFD, *n* = 1).

### 3.2. Pharmacological Compounds Increasing the Acetylcholine Neurotransmission

To modulate acetylcholine neurotransmission, seven pharmacological compounds with different mechanisms of actions have been tested, including AChE inhibitors, inhibitors of acetylcholine exocytosis, and acetylcholine precursors (Table 1). All these compounds act to increase the quantity of acetylcholine in the synaptic cleft.

AChE inhibitors were the most commonly investigated compounds, such as ambenonium chloride, donepezil, huperzine-A, neostigmine, and physostigmine. All these compounds have been tested in the three animal models of PNP. Most of the compounds induced antinociceptive effects, both on mechanical and thermal hypersensitivity [20,31,32,33,34,35,36,37]. A low dose of neostigmine (intrathecal injection, 0.3 ng) did not produce significant antinociceptive effects [38].

Botulinum neurotoxin type A has been used to inhibit the exocytosis of acetylcholine and has shown a persistent antinociceptive effect on mechanical hypersensitivity [39,40].

Finally, citicoline, a precursor of choline, exhibited antinociceptive properties on mechanical hypersensitivity after intracerebroventricular injection or nerve application [41,42,43].

**Table 1:** Description of studies assessing pharmacological compounds modulating the acetylcholine neurotransmission.

Compounds	Targets	Doses, Routes	Models	Species (Sex)	Behavioral Assays	Effects	Ref.
Ambenonium chloride	AChE inhibitors	0.05 mg/kg, i.p.	Traumatic	Mouse (♂)	Von Frey hairs Conditioned place preference	↘ M 0 spontaneous pain	[34]
Donepezil		0.3–1 mg/kg, i.p.	Traumatic	Rat (♂)	Paw pressure	↘ M	[20]
		0.3–1.0 mg/kg, i.p.	Traumatic	Rat (♂)	Paw pressure	Dose-dependent ↘ M (0.6 and 1 mg/kg)	[31]
		5–10 mg/kg, p.o.	CIPN + traumatic	Rat (♂)	Electronic Von Frey Paw immersion 10 °C and 46 °C	↘ M + T	[32]
		5 mg/kg, p.o.	CIPN	Rat (♂)	Electronic Von Frey Tail immersion 10 °C	↘ M + T	[33]
Huperzine-A		0.1–0.15 mg/kg, i.p.	Traumatic	Mouse (♂)	Von Frey hairs Conditioned place preference	↘ M 0 spontaneous pain	[34]
Neostigmine		2 mg, i.t.	Traumatic	Rat (♂)	Von Frey hairs	↘ M	[36]
		0.3–3 ng, i.t.	Traumatic	Mouse (♂)	Plantar test Von Frey hairs	Dose-dependant ↘ M + T (3 ng)	[38]
		0.1–0.5 µg, i.t.	Diabetic	Rat (♂)	Von Frey hairs	Dose-dependent ↘ M (0.5 µg)	[37]
Physostigmine		15 nmol, i.t.	Traumatic	Mouse (♂)	Von Frey hairs	↘ M	[35]
BoNT/A	Acetylcholine exocytosis inhibitors	15 pg, i.p.	Traumatic	Mouse (♂)	Electronic Von Frey	↘ M	[39]
BoNT/A Dysport^®^ BoNT/A Botox^®^		20 U/kg, s.p.	CIPN	Rat (♂)	Paw pressure	↘ M	[40]
Citicoline	Acetylcholine precursor	0.4–0.8 mL (100 µmol/L), p.n.	Traumatic	Rat (♂)	Von Frey hairs	↘ M	[41]
		0.5–2 µmol, i.c.v.	Traumatic	Rat (♂)	Paw pressure	Dose- and time-dependent ↘ M (1 and 2 µmol)	[42]
		0.5–2 µmol, i.c.v.	CIPN	Rat (♂)	Paw pressure	Dose- and time-dependent ↘ M (1 and 2 µmol)	[43]

BoNT/A: botulinum neurotoxin type A; AChE: acetylcholine esterase; p.o.: per os; i.p.: intraperitoneal; i.t.: intratechal; p.n.: perinervous; s.p.: subplantar; i.c.v.: intracerebroventricular; ↘: decrease; 0: no effect; M: mechanical sensitivity; T: thermal sensitivity; ♀: female; ♂: male.

### 3.3. Pharmacological Compounds Targeting Nicotinic Acetylcholine Receptors

#### 3.3.1. Unspecific Targeting of Nicotinic Acetylcholine Receptors

Four compounds unspecifically targeting nAChRs were identified: epibatidine, nicotine, S(−)-nornicotine, and R(+)-nornicotine (Table 2).

Nicotine was the most studied compound at various doses and routes (1–30 nmol i.t., 0.1–2 mg/kg s.c., and 0.3–1.75 mg/kg i.p.), mainly in traumatic PNP models, with one study on a CIPN model (paclitaxel). In several publications, nicotine demonstrated analgesic properties on mechanical and thermal hypersensitivity. It showed a decrease in mechanical allodynia in a time- and dose-dependent manner [18,44,45,46,47,48,49]. However, chronic administration of nicotine (24 mg/kg/day, osmotic pump) induced a dose-dependent and stable mechanical hypersensitivity [50]. Similarly, repeated administrations of nicotine induced a decrease in its analgesic effects (heat hypersensitivity) [51]. Other studies have also reported a reduction in mechanical and thermal hypersensitivity [52,53]. Two enantiomers, S(−)-nornicotine and R(+)-nornicotine, dose-dependently reversed mechanical hypersensitivity [54].

Finally, epibatidine has also been found to dose-dependently reverse mechanical allodynia in traumatic PNP [18,48,55].

**Table 2:** Description of studies assessing pharmacological compounds targeting nicotinic acetylcholine receptors.

Compounds	Targets	Doses, Routes	Models	Species (Sex)	Behavioral Assays	Effects	Ref.
Epibatidine	nAChRs agonists	0.03–0.3 nmol, i.t.	Traumatic	Mouse (♂)	Electronic Von Frey Plantar test	Dose-dependent ↘ M (all doses effective)	[18]
		0.3–10.0 µg/kg, s.c.	Traumatic	Rat (♂)	Paw pressure	Dose-dependent ↘ M (all doses effective)	[55]
		0.036–0.36 pmol, i.t.	Traumatic	Rat (♂)	Paw pressure	Dose-dependent ↘ M	[48]
Nicotine		0.1–1.5 mg/kg, s.c.	Traumatic	Mouse (♂)	Von Frey hairs	Dose- and time-dependent↘ M (1 and 1.5 mg/kg)	[45]
		0.1–10 nmol, i.t.	Traumatic	Rat (♂)	Electronic Von Frey	Dose- and time-dependent↘ M (1 nmol)	[46]
		3–30 nmol, i.t.	Traumatic	Mouse (♂)	Electronic Von Frey Plantar test	Dose-dependent ↘ M (10 and 30 nmol)	[18]
		2.2–6.5 nmol, i.t.	Traumatic	Rat (♂)	Paw pressure	Dose-dependent ↘ M	[48]
		0.25–1.75 mg/kg, i.p.	Traumatic	Mouse (♂ + ♀)	Von Frey hairs	Dose-dependent ↘ M	[49]
		0.25–25 µg, i.c.v.	Traumatic	Mouse (♂ + ♀)	Von Frey hairs	Dose-dependent ↘ M	[49]
		0.25–17.5 µg, i.t.	Traumatic	Mouse (♂ + ♀)	Von Frey hairs	Dose-dependent ↘ M	[49]
		25–100 µg, i.pl.	Traumatic	Mouse (♂ + ♀)	Von Frey hairs	Dose-dependent ↘ M	[49]
		4 or 10 mg/kg/day, s.c.	Traumatic	Rat (♂)	Paw pressure	↗ M	[50]
		20 nmol/day/4 days, p.n. 1–20 nmol, p.n.	Traumatic	Mouse (♂)	Von Frey hairs Plantar test	↘ M (preventive effect) Dose-dependent ↘ M + T (5 and 20 nmol)	[47]
		2 mg/kg, s.c.	Traumatic	Mouse (♂)	Von Frey hair	↘ T	[51]
		1 mg/kg, i.v.	Traumatic	Mouse (♂)	Von Frey hairs Plantar test Tail-flick	↘ M + T	[52]
		20 nmol, p.n.	Traumatic	Mouse (♂)	Von Frey hair Plantar test	↘ M + T	[53]
		0.3–0.9 mg/kg, i.p. 24 mg/kg/day, s.c.	CIPN	Mouse (♂)	Von Frey hairs	Dose-dependent ↘ M (0.6 and 0.9 mg/kg) ↘ M (preventive effect)	[44]
S(-)-nornicotine		5–20 mg/kg, i.p.	Traumatic	Rat (♂)	Paw pressure	Dose-dependent ↘ M (20 mg/kg)	[54]
R(+)-nornicotine		10–15 mg/kg, i.p.	Traumatic	Rat (♂)	Paw pressure	Dose-dependent ↘ M (15 mg/kg)	[54]

i.p.: intraperitoneal; i.t.: intratechal; s.c.: subcutaneous; i.c.v.: intracerebroventricular; i.pl.: intraplantar; p.n.: perinervous; ↘: decrease; ↗: increase; M: mechanical sensitivity; T: thermal sensitivity; ♀: female; ♂: male.

#### 3.3.2. Specific Targeting of α4β2 Nicotinic Acetylcholine Receptors

Ten compounds specifically targeting of α4β2 nAChRs have been identified. Nine of them were agonists ([123/125I]5IA, A-366833, A-85380, NS9283, ABT-418, ABT-594, Bee venom, C-9515, RJR-2403, and TC-2559 [46,55,56,57,58,59,60,61,62,63,64,65,66]), and one a partial agonist (Sazetidine A [56]) (Table 3). These compounds were primarily tested in traumatic PNP models.

All the tested compounds produced antinociceptive effects, and mostly on mechanical hypersensitivity. However, NS9283 and A-85380 (administered at low pmol dose intrathecally) did not show any effects on mechanical or thermal hypersensitivity in traumatic PNP [48,67].

**Table 3:** Description of studies assessing pharmacological compounds targeting α4β2 nicotinic acetylcholine receptors.

Compounds	Targets	Doses, Routes	Models	Species (Sex)	Behavioral Assays	Effects	Ref.
[123/125I]5IA	α4β2 nAChR agonists	1–100 nmol, i.c.v.	Traumatic	Rat (♂)	Von Frey hairs	Dose-dependent ↘ M (50 and 100 nmol)	[57]
		1–10 nmol, i.c.v.	Traumatic	Rat (♂)	Von Frey hairs	Dose-dependent ↘ M (3 and 10 nmol)	[63]
A-366833		1.9–19 µmol/kg, i.p.	Traumatic	Rat (♂)	Von Frey hairs	Dose-dependent ↘ M (all doses effective)	[64]
		1–6 mg/kg, i.p.	Traumatic + Diabetic +CIPN	Rat (♂)	Paw pressure	↘ M	[65]
A-85380		0.125–1 µmol/kg, i.p.	Traumatic	Rat (♂)	Von Frey hairs	Dose-dependent ↘ M (0.75 and 1 µmol/kg	[66]
		<pmol, i.t.	Traumatic	Rat (♂)	Paw pressure	0 M	[48]
NS9283		35 mmol/kg, i.p.	Traumatic	Rat (♂)	Von Frey hair	0 M	[67]
ABT-418		1–20 nmol, p.n.	Traumatic	Mouse (♂)	Von Frey hairs Plantar test	↘ M + T	[53]
ABT-594		3–100 µg/kg, s.c.	Traumatic	Rat (♂)	Von Frey hairs	Dose-dependent ↘ M (10–100 µg/kg)	[55]
		0.01–0.3 µmol/kg, i.p.	CIPN	Rat (♂)	Von Frey hairs	Dose-dependent ↘ M (0.1–0.3 µmol/kg)	[59]
Bee venom		0.25 mg/kg, s.c.	CIPN	Rat (♂)	Tail immersion test	↘ T	[61]
C-9515		0.001–0.01 mg/kg, i.p.	Traumatic	Rat (♂)	Von Frey hairs	Dose-dependent ↘ M (0.003 and 0.01 mg/kg)	[58]
RJR-2403		1–100 nmol, i.t.	Traumatic	Rat (♂)	Electronic Von Frey	↘ M	[46]
TC-2559		2.28–22.8 µmol/kg, s.c. 20 nmol, p.n.	Traumatic	Mouse (♂)	Von Frey hairs	Dose-dependent ↘ M (22.8 µmol/kg) ↘ M	[56]
		1–20 nmol, p.n.	Traumatic	Mouse (♂)	Von Frey hairs Plantar test	↘ M + T	[53]
		0.3–3 mg/kg, i.p.	Traumatic	Rat (♂)	Von Frey hairs	Dose-dependent ↘ M (1 and 3 mg/kg)	[62]
		20 nmol, p.n. 10 mg/kg, s.c.	Diabetic	Mouse (♂)	Von Frey hair	↘ M	[60]
Sazetidine A	α4β2 nAChR partial agonist	0.2–20 nmol, p.n.	Traumatic	Mouse (♂)	Von Frey hairs	Dose-dependent ↘ M (20 nmol)	[56]

i.p.: intraperitoneal; i.t.: intratechal; s.c.: subcutaneous; i.c.v.: intracerebroventricular; p.n.: perinervous; s.c.: subcutaneous; ↘: decrease; 0: no effect; M: mechanical hypersensitivity; T: thermal hypersensitivity; ♀: female; ♂: male.

#### 3.3.3. Specific Targeting of α7 Nicotinic Acetylcholine Receptors

Seventeen compounds targeting α7 nAChRs were identified. Among them, ten were α7 nAChR agonists, two were α7 nAChR silent agonists, three were positives allosteric modulators (PAM) of α7 nAChRs, one was an α3β4 and α7 nAChRs antagonist, and one was an α7 nAChRs + m4AChRs agonist (Table 4). These compounds were mostly tested in traumatic PNP models.

Among the α7 nAChR agonists, all the tested compounds ((R)-ICH3, choline, cobratoxin, DM489, GAT107, PAM-4, PHA-543613, PNU-28298, PNU-282987, and TC-7020) exhibited antinociceptive effects on mechanical and thermal hypersensitivity [46,68,69,70,71,72,73,74,75,76]. While GAT107 was effective on mechanical hypersensitivity, no effect was reported on thermal hypersensitivity [72].

The two α7 nAChR silent agonists, NS6740 and R-47, demonstrated antinociceptive effects on mechanical hypersensitivity [77,78].

Regarding the three PAM of α7 nAChRs, only PAM-2 and PNU-120596 had analgesic effects [69,73,79], but NS1738 had no effect on mechanical and thermal hypersensitivity [69].

The α3β4 and α7 nAChRs antagonist, α-conopeptides Eu1.6 [80], and the α7 nAChRs + m4AChRs agonist, DXL-A-24 [81], both decreased mechanical hypersensitivity.

**Table 4:** Description of studies assessing pharmacological compounds targeting α7 nicotinic acetylcholine receptor.

Compounds	Targets	Doses, Routes	Models	Species (Sex)	Behavioral Assays	Effects	Ref.
(R)-ICH3	α7 nAChR agonist	30 mg/kg, p.o.	CIPN	Rat (♂)	Paw pressure Von Frey hairs Cold plate (4 °C)	↘ M + T	[70]
Choline		100 nmol, i.t.	Traumatic	Rat (♂)	Electronic Von Frey	↘ M	[46]
Cobratoxin		0.56–4.5 µg/kg, i.t.	Traumatic	Rat (♂)	Paw pressure Tail-flick	Dose-dependent ↘ M + T (1.12 and 4.5 µg/kg)	[68]
DM489		3–10 mg/kg, p.o.	Diabetic	Mouse (♂)	Cold plate 4 °C	↘ T	[73]
		10–30 mg/kg, p.o.	CIPN	Mouse (♂)	Cold plate 4 °C	↘ T	[73]
GAT107		1–10 mg/kg, i.p.	Traumatic	Mouse (♂)	Von Frey hair Plantar test	Dose- and time-dependent ↘ M (3 and 10 mg/kg) ↘ T	[72]
PAM-4		1–2 mg/kg, i.p.	Traumatic	Mouse (♂)	Von Frey hairs	Dose- and time-dependent ↘ M (2 mg/kg)	[74]
PHA-543613		12 μg, i.t.	Traumatic	Rat (♂)	Electronic von frey Plantar test	↘ M + T	[71]
		1–6 mg/kg, s.c.	Traumatic	Mouse (♂)	Von Frey hairs	Dose-dependent ↘ M (6 mg/kg)	[69]
PNU-28298		1–30 mg kg, p.o.	Traumatic	Rat (♂)	Paw pressure	Dose-dependent ↘ M	[75]
PNU-282987		30 mg/kg, p.o.	CIPN	Rat (♂)	Paw pressure	↘ M	[70]
		1 µg/kg, i.t.	Traumatic	Rat (♂)	Paw pressure Tail-flick	↘ M + T	[68]
TC-7020		1–10 mg/kg/day, s.c.	Traumatic	Rat (♂)	Von Frey hairs	↘ M	[76]
NS6740	α7 nAChR silent agonist	1–9 mg/kg, i.p.	Traumatic	Mouse (♂)	Von Frey hairs	Dose-dependent ↘ M (9 mg/kg)	[77]
R-47		0–10 mg/kg, p.o.	CIPN	Mouse (♂)	Von Frey hairs	Dose- and time-dependent ↘ M (5 and 10 mg/kg)	[78]
PAM-2	α7 nAChR PAM	3 mg/kg, p.o.	Diabetic + CIPN	Mouse (♂)	Cold plate 4 °C	↘ T	[73]
		2–8 mg/kg, i.p.	Traumatic	Mouse (♂)	Von Frey hairs	Dose-dependent ↘ M (6 and 8 mg/kg)	[79]
NS1738		30 mg/kg, i.p.	Traumatic	Mouse (♂)	Von Frey hairs Plantar test	0 M + T	[69]
PNU-120596		1–8 mg/kg, i.p.	Traumatic	Mouse (♂)	Von Frey hairs Plantar test	↘ M + T	[69]
α-conopeptides Eu1.6	α3β4/α7 nAChR antagonist	0.5–24.9 μg/kg, i.m.	Traumatic	Rat (♂)	Paw pressure	↘ M	[80]
DXL-A-24	α7 nAChR/ m4AChR agonist	0.25–1 mg/kg, p.o. daily	Traumatic	Rat (♂)	Von Frey hairs Hot plate 50 °C	Dose- and time-dependent ↘ M + T (0.5 and 1 mg/kg)	[81]

PAM: positive allosteric modulator; p.o.: per os; i.p.: intraperitoneal; i.m.: intramuscular; i.t.: intratechal; s.c.: subcutaneous; ↘: decrease; 0: no effect; M: mechanical sensitivity; T: thermal sensitivity; ♀: female; ♂: male.

#### 3.3.4. Specific Targeting of α9/α10 Nicotinic Acetylcholine Receptors

We identified seventeen α9/α10 nAChR antagonists (Table 5). These compounds have been tested in traumatic and CIPN PNP models, but not in diabetic models. 

Most of the studies explored α-conotoxins and derivatives as potent antagonists of α9/α10 nAChR, including α-conotoxin AuIB, α-conotoxin MII, α-conotoxin Mr1.1 [S4Dap], α-conotoxin RgIA, α-conotoxin RgIA4, α-conotoxin RgIA-5474, [2,8]-alkyne Vc1.1 3, GeXIVA[1,2], GeXIVA[1,4], α-conotoxin Vc1.1, Vc1.1[N9R], and [P6O]Vc.1.1. Doses ranged from 0.036 to 60 µg (i.m. route), from 0.128 to 80 µg/kg (s.c. route), and from 0.02 to 2 nmol (i.t. route).

Nearly all the α9/α10 nAChR antagonists, including (±)-18-MC, (+)-catharanthine, α-conotoxins AuIB, α-conotoxins MII, α-conotoxins Mr1.1 [S4Dap], α-Conotoxin RgIA, α-conotoxins RgIA4, α-Conotoxin RgIA-5474, [2,8]-alkyne Vc1.1 3, α-conotoxins Vc1.1, Vc1.1[N9R], GeXIVA[1,2], GeXIVA[1,4], ZZ-204G, and ZZ1-61c, presented antinociceptive effects on mechanical and thermal hypersensitivity [80,82,83,84,85,86,87,88,89,90,91,92,93,94,95,96,97,98,99,100,101,102,103,104]. [P6O]Vc.1.1 had no effect on mechanical hypersensitivity [85], while α-conotoxins RgIA4 (assessed in paclitaxel-related CIPN model) [98], GeXIVA[1,2] (assessed in oxaliplatin-related CIPN model) [102], and ZZ-204G (assessed with the tail flick test in naïve animals) [89], had no effects on thermal hypersensitivity.

**Table 5:** Description of studies assessing pharmacological compounds targeting α9/α10 nicotinic acetylcholine receptors.

Compounds	Targets	Doses, Routes	Models	Species (Sex)	Behavioral Assays	Effects	Ref.
(±)-18-MC	α9/α10 nAChR antagonist	72 mg/kg, p.o.	CIPN	Mouse (♂)	Cold plate 4 °C	↘ T	[90]
(+)-catharanthine		36–72 mg/kg, p.o.	CIPN	Mouse (♂)	Cold plate 4 °C	↘ T	[90]
α-conotoxin AuIB		0.02–2 nmol, i.t.	Traumatic	Rat (♂)	Von Frey hairs	Dose-dependent ↘ M (2 nmol)	[82]
		0.36–36 µg, i.m.	Traumatic	Rat (♂)	Von Frey hairs	↘ M	[83]
α-conotoxin MII		0.02–2 nmol, i.t.	Traumatic	Rat (♂)	Von Frey hairs	Dose-dependent ↘ M (2 nmol)	[82]
		0.36–36 µg, i.m.	Traumatic	Rat (♂)	Von Frey hairs	↘ M	[83]
α-conotoxin Mr1.1 [S4Dap]		0.5–25 µg/kg, nr	Traumatic	Rat (♂)	Electronic Von Frey	Dose-dependent ↘ M (25 µg/kg)	[91]
α-conotoxin RgIA		2–10 nmol, i.m.	Traumatic	Rat (♂)	Paw pressure Electronic Von Frey	↘ M	[84]
α-conotoxin RgIA4		0.02–0.2 nmol, i.m.	Traumatic	Rat (♂)	Paw pressure	Dose-dependent ↘ M (0.2 nmol)	[92]
		100 mg/kg, i.m.	CIPN	Mouse (♂)	Hot plate 47 °C Electronic Von Frey Acetone test	↘ T + M	[93]
		2–10 nmol, i.m.	CIPN	Rat (♂)	Cold plate 4 °C	↘ T	[94]
		0.128–80 µg/kg, s.c.	CIPN	Mouse + Rat (♂)	Paw pressure Cold plate 4 °C	Dose-dependent ↘ M + T (all doses effective)	[95]
		40 µg/kg, s.c.	CIPN	Mouse (nr)	Cold plate (decrease of 10 °C/min)	↘ T	[96]
		40 µg/kg, s.c.	CIPN	Mouse (nr)	Cold plate (decrease of 10 °C/min)	↘ T	[97]
		16–80 µg/kg, s.c.	CIPN	Rat (♂)	Von Frey hairs Cold plate 5 °C Plantar test	Time-dependent ↘ M 0 T	[98]
α-conotoxin RgIA-5474		4–40 µg/kg, s.c.	CIPN	Mouse (nr)	Cold plate (decrease of 10 °C/min)	↘ T	[99]
α-conotoxin Vc1.1		0.02–2 nmol, i.t.	Traumatic	Rat (♂)	Von Frey hairs	Dose-dependent ↘ M (2 nmol)	[82]
		0.36–36 µg, i.m.	Traumatic	Rat (♂)	Von Frey hairs	Dose-dependent ↘ M (all doses effective)	[83]
		0.36–3.6 µg, i.m.	Traumatic	Rat (♂)	Paw pressure	Dose-dependent ↘ M (3.6 µg)	[101]
		0.036–0.36 µg, i.m.	Traumatic	Rat (♂)	Paw pressure	Dose-dependent ↘ M	[92]
		60 µg, i.m.	Traumatic	Rat (♂)	Von Frey hairs	↘ M	[85]
		27.2–54.2 μg/kg, i.m.	Traumatic	Rat (♂)	Paw pressure	↘ M	[80]
[2,8]-alkyne Vc1.1 3		0.03–0.1 mg/kg, i.m.	Traumatic	Rat (♂)	Von Frey hairs	Dose-dependent ↘ M (0.1 mg/kg)	[100]
Vc1.1[N9R]		0.3–15 nmol/kg, i.m.	Traumatic	Rat (♂)	Paw pressure	↘ M	[86]
[P6O]Vc.1.1		60 µg, i.m.	Traumatic	Rat (♂)	Von Frey hairs	0 M	[85]
GeXIVA[1,2]		0.5–2 nmol, i.m.	Traumatic	Rat (♂)	Electronic Von Frey	Dose-dependent ↘ M (All doses effective)	[104]
		0.3–1.2 nmol, i.m.	Traumatic	Rat (♂)	Electronic Von Frey Von Frey hairs	↘ M	[87]
		32–128 nmol/kg, i.m.	CIPN	Rat (♂)	Von Frey hairs + acetone test + tail-flick	Dose-dependent ↘ (128 nmol/kg) 0 T	[102]
		0.45 mg/kg, i.m.	CIPN	Rat (♂)	Von Frey hairs Tail-flick	↘ M + T	[103]
GeXIVA[1,4]		0.5–2 nmol, i.m.	Traumatic	Rat (♂)	Electronic Von Frey	Dose-dependent ↘ M (1 and 2 nmol)	[104]
Oligoarginine R8		20 mg/kg, i.m.	CIPN	Mouse (♂)	Hot plate 47 °C Electronic Von Frey Acetone test	↘ M + T	[93]
ZZ-204G		3.6–3600 µg/kg, i.p.	Traumatic	Rat (♂)	Paw pressure Tail-flick	Dose-dependent ↘ M (360 and 3600 µg/kg) 0 T	[89]
ZZ1-61c		100 µg/kg/day, i.p.	CIPN	Rat (♂)	Paw pressure	↘ M	[88]

p.o.: per os; i.p.: intraperitoneal; i.m.: intramuscular; i.t.: intratechal; s.c.: subcutaneous; nr: not reported; ↘: decrease; 0: no effect; M: mechanical hypersensitivity; T: thermal hypersensitivity; ♀: female; ♂: male.

### 3.4. Pharmacological Compounds Targeting Muscarinic Acetylcholine Receptors

Eight compounds targeting mAChRs have been identified, including one mAChR non-selective agonist (oxotremorine), one mAChR antagonist (scopolamine), one agonist of m2/m4AChRs and antagonist of m1/m3/m5AChRs (PTAC), one m4/α7AChRs agonist (DXL-A-24), one m1/m4AChRs agonist (McNA-343), one m1AChR agonist (PPBI), and two m1AChR antagonists (pirenzepine and VU0255035) (Table 6). These compounds were mostly tested in traumatic PNP models.

All these compounds induced antinociceptive effects in PNP models and mostly on mechanical hypersensitivity [38,81,105,106,107,108,109,110]. Only McNA-343 (m1/m4AChRs agonist) did not alleviate thermal hypersensitivity [38]. Scopolamine (mAChRs antagonist) injected in the anterior cingulate cortex decreased the autotomy behavior of animals (sciatic nerve section) [107].

Surprisingly, both agonist (PPBI) and antagonists (pirenzepine and VU0255035) of m1AChRs demonstrated antinociceptive effects in PNP models [109,110].

**Table 6:** Description of studies assessing pharmacological compounds targeting muscarinic acetylcholine receptors.

Compounds	Targets	Doses, Routes	Models	Species (Sex)	Behavioral Assays	Effects	Ref.
Oxotremorine	mAChRs non-selective agonist	5–10 μg, i.t.	Traumatic	Rat (♂)	Von Frey hairs Ethyl chloride spray Plantar test	↘ M + T	[105]
PTAC	m2-4 AChRs agonist m1-3-5 AChRs antagonist	0.05–0.1 mg/kg, i.p.	Traumatic	Mouse (♂)	Von Frey hairs	↘ M	[106]
Scopolamine	mAChRs antagonist	0.4 μg/μL, ACC	Traumatic	Rat (♂)	Daily autotomy scores	↘ autotomy	[107]
DXL-A-24	m4AChR + α7 nAChR agonist	0.25–1 mg/kg, p.o.	Traumatic	Rat (♂)	Von Frey hairs	Dose-dependent ↘ M (0.5 and 1 mg/kg)	[81]
McNA-343	m1/m4AChRs agonist	18.9–1890 pmol, ACC	Traumatic	Rat (♂)	Electronic Von Frey	Dose-dependent ↘ M (189 and 1890 pmol)	[108]
		3–10 μg, i.t.	Traumatic	Mouse (♂)	Plantar test Von Frey hairs Paw pressure	↘ M 0 T	[38]
PPBI	m1AChR agonist	0.2–100 mmol/kg, p.o.	Traumatic + CIPN	Mouse + Rat (♂)	Tail immersion 13 °C Acetone test Plantar test	Dose-dependent ↘ M + T	[109]
Pirenzepine	m1AChR antagonist	10 mg/kg/d, s.c.	Diabetic + CIPN	Mouse + Rat (♂ + ♀)	Von Frey hairs	↘ M	[110]
VU0255035		10 mg/kg, i.p.	Diabetic	Mouse + Rat (♂ + ♀)	Von Frey hairs	↘ M	[110]

i.p.: intraperitoneal; i.t.: intratechal; s.c.: subcutaneous; p.o.: per os; ACC: anterior cingulate cortex injection; ↘: decrease; 0: no effect; M: mechanical sensitivity; T: thermal sensitivity; ♀: female; ♂: male.

## 4. Discussion

This review aimed to assess the state of the art regarding compounds targeting acetylcholine neurotransmission for the management of PNP in rodent models.

Among the 82 selected publications, 62 cholinergic compounds were assessed in rodent models of PNP. Almost all the compounds had antinociceptive effects in the PNP models, demonstrating the interest in targeting acetylcholine neurotransmission for pain management. Most of the studies assessed the compounds using traumatic models of PNP, and to a lesser extent, in CIPN or diabetic models. Two third of the studies were conducted with rats, and one third with mice. Most of the studies utilized male animals, and only two studies employed both male and female animals. However, sex differences exist in the context of PNP, particularly concerning the sexually dimorphic nature of neuroimmune pathophysiology [111], which highlights the importance of including subjects of both sexes in preclinical pain research [112].

In two-thirds of the assays, compounds were administered via systemic routes, with the i.p. route accounting for 23.2%, the i.m. route for 18.8%, the s.c. route for 13.6%, and the p.o. route for 11.6%. The remaining third of the assays employed local routes of administration, with the most representative ones being the i.t. route for 18.8%, p.n. for 5.4%, and the i.c.v. route for 4.5%. In spite of the interesting use of local administrations for defining precise mechanisms of action, systemic routes should be encouraged to improve the translationality of the results and drugability of the compounds. The assessment of the analgesic effects of compounds was primarily conducted using tests exploring mechanical hypersensitivity, which accounted for approximately 75% of the tests. Thermal hypersensitivity was less explored, comprising approximately 25% of the tests. Interestingly, in some cases, the same compound demonstrated effectiveness on mechanical hypersensitivity but not on thermal hypersensitivity (e.g., the α7 nAChR agonist GAT107 [72]; the α9/α10 nAChR antagonists α-conotoxin RgIA4 [98], GeXIVA[1,2] [84], and ZZ-204G [89]; and the m1/m4AChRs agonist McNA-343 [38]). Therefore, the exploration of the analgesic activity of these compounds should be encouraged on both modalities (mechanical and thermal) to better define their effects.

**Figure 2:**
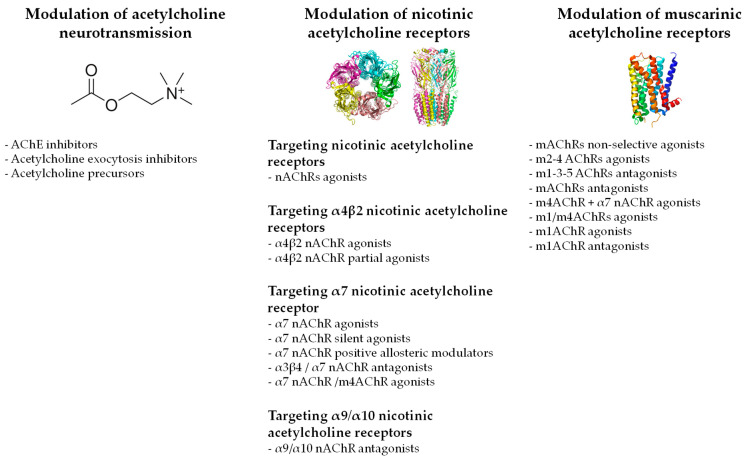
Pharmacological targets identified in the selected publications (image: Wikipedia).

The present review highlights that research has primarily focused on nAChRs compared with mAChRs (Figure 2). Among the nAChRs, the α4β2 nAChR and α7 nAChR agonists, and α9/α10 nAChR antagonists were the main compounds explored. The first approach was driven by a global activation of nAChRs, thanks to nicotine [18,44,45,46,47,48,49,50,51,52,53]. Epibatidine, a toxic alkaloid isolated and identified from *Epipedobates tricolor* skin (for a review see [113]), was also used in a similar approach [18,48,55], and even though this compound is recognized today as an α4β2 nAChR agonist, it is also able to target α7 nAChR and α3β4 nAChR [113]. A more specific targeting of nAChRs has been tested with specific agonists of α4β2 nAChR and α7 nAChR, and antagonists of α9/α10 nAChR. The latter were mainly focused on α-conotoxins and derivatives originating from venomous marine cone snails (*Conus species*) [114].

α4β2 nAChR stands out as the most prevalent heteromeric subtype within the brain. Typically labeled as α4β2* nAChR, the asterisk acknowledges the potential involvement of additional subunits. When presented in isolation, the α4 and β2 subunits coalesce to form two distinctive functional isoforms: the low-sensitivity (α4)3(β2)2 and high-sensitivity (α4)2(β2)3 nAChRs. Notably, the lower acetylcholine sensitivity of (α4)3(β2)2 nAChR constitutes the majority within the cortex [115]. This α4β2* nAChR variant assumes a crucial role in nicotine dependence and represents a pharmacological target for smoking cessation aids like varenicline [115].

In contrast, the homopentameric α7 nAChR lacks the synaptic functional adaptations observed in muscle-type and ganglionic nAChRs. The absence of non-alpha subunits influences the agonist binding site, shifting high-affinity agonist binding toward desensitized states and enhancing sensitivity to the widely available acetylcholine precursor, choline [116]. Unique cellular and subcellular distribution patterns for α7 nAChR indicate its distinct roles. These distinctive patterns encompass widespread expression in non-neuronal cells, including those within the immune system. Notably, α7 nAChR plays a distinctive role in regulating the cholinergic anti-inflammatory pathway. An intriguing attribute of open α7 nAChR is its heightened calcium permeability [116]. Although this characteristic could potentially lead to excitotoxicity akin to NMDA-type glutamate receptors, the normally low open probability of the α7 nAChR receptor channel offsets this risk [116]. It is noteworthy that modifications in intracellular calcium concentration following α7 nAChR stimulation predominantly arise from the release of calcium stored intracellularly rather than calcium influx through α7 nAChR channels, implying a metabotropic-like effect [116].

α9/α10 nAChR represents a more recently studied subtype. Its primary functions have been elucidated in inner ear mechanosensory hair cells. Considered a non-neuronal nAChR, the α9/α10 subtype demonstrates negligible expression of α9 and very low expression of α10 subunits in murine DRG [12,117]. However, in human DRG, around 25% of sensory neurons have been found to express α9 and α10 mRNA [117]. Notably, the α9/α10 nAChR holds potential as an intriguing target for inner ear disorders from a pharmacotherapeutic perspective [12]. Beyond auditory disorders, blocking α9/α10 nAChRs has displayed analgesic effects linked to the modulation of immune cells and inflammatory processes [13].

Excluding α9/α10 nAChR, nAChRs are distributed across neurons in peripheral nociceptive nerve fibers, DRG, the dorsal horn of the spinal cord, the RVM in the brainstem, and the periaqueductal gray, all of which play pivotal roles in pain perception and processing [118]. Moreover, α4β2 nAChRs have been identified within astrocytes in the spinal cord and regions of the brain associated with pain processing [119]. α7 nAChRs display expression in immune and non-immune cells responsible for cytokine production, exercising control over immune cell functions, suppressing the production of pro-inflammatory cytokines, and mitigating inflammatory processes [116].

Finally, the targeting of mAChRs was clearly less explored, with few compounds and a widespread targeting of both agonists and antagonists for specific and unspecific mAChRs (Figure 2). No specific mAChRs emerged as clear pharmacological targets. Moreover, the definition of the compounds’ activities can be obscure. For example, both agonists and antagonists of the same m1AChRs induced antinociceptive effects [109,110], which raises doubt about the selectivity of these compounds. This targeting of mAChRs for pain management remains clearly underexplored, and more research is need.

Another interesting point is that compounds modulating the acetylcholine neurotransmission (e.g., AChE inhibitors, acetylcholine exocytosis inhibitors, and acetylcholine precursors), which ultimately increase the quantity of acetylcholine in the synaptic cleft, demonstrated analgesic effects (Figure 2) [20,31,32,33,34,35,36,37,39,40,41,42,43]. This unspecific strategy stands in stark contrast to the targeting of specific AChRs for pain modulation described in this review. By increasing the quantity of acetylcholine, these compounds promote global activation of nAChRs and mAChRs, which can be opposite to the sought effect in the case of α9/α10 nAChRs, where an inhibition of the latter is needed for an analgesic effect [120].

Studies on nicotine have explored the desensitization of its activity after chronic exposure, showing a decrease in its antinociceptive effects and even nociceptive sensitization, mediated by a desensitization of α4β2 nAChRs [50]. This desensitization was related to an increase in the phosphorylation of cAMP response element-binding protein (pCREB) in the spinal cord, which is highly correlated with the degree of mechanical hypersensitivity [50]. It is well demonstrated that tobacco smoking has acute antinociceptive effects [121] and chronic pronociceptive ones [122]. Tobacco smoking is a risk factor for chronic pain [122]. This desensitization of nAChRs has also been reported for α7 nAChR and α3β4 nAChR, driving the research from agonists of specific nAChRs to positive allosteric modulators of these nAChRs. Positive allosteric modulators can increase the binding affinity and/or efficacy of an orthosteric agonist (endogenous or exogenous) [123].

To our knowledge, besides BoNT/A, which is mainly authorized for various spasticity disorders, no cholinergic compound is currently authorized for the clinical management of PNP. The use of cholinergic compounds in patients experiencing pain could raise safety concerns regarding the cholinergic mechanism of action. We can extrapolate adverse effects of these compounds from those of AChE inhibitors used in Alzheimer’s disease treatment and medications used for smoking cessation (nicotine replacement therapy and varenicline). The most frequently reported adverse events of AChE inhibitors are gastrointestinal issues, including nausea, vomiting, diarrhea, and anorexia [124]. In the case of nicotine replacement therapy, the most commonly reported adverse events include headache, dizziness/light-headedness, nausea/vomiting, gastrointestinal symptoms, sleep/dream problems, and cardiovascular effects (palpitations, chest pain) [125]. With varenicline, the most frequently reported adverse events are nausea, insomnia, abnormal dreams, and headache [126].

### Limitations of This Review

The aim of this review was to present an overview of the current studies focused on compounds that modulate cholinergic neurotransmission in the management of PNP in rodent models. This aim was not to report effective compounds on PNP. However, we believe that there is still a publication bias related to the fact that many negative results have not been published [127], and consequently, several studies focused on compounds that modulate cholinergic neurotransmission would be underreported.

## 5. Conclusions

The objective of this scoping review was to identify the primary targets and compounds studied in animal models of PNP. Besides mAChRs, nAChRs remain the most extensively investigated targets, with specific compounds such as α4β2 nAChR and α7 nAChR agonists, as well as α9/α10 nAChR antagonists. Notably, for the latter group, a majority of studies have focused on conotoxins and derivatives, representing innovative compounds. In contrast, when it comes to mAChRs, no distinct cholinergic target can be definitively defined based on this review.

The next steps involve a clear delineation of the antinociceptive mechanisms of action, including a more precise identification of the targeted AChRs and the cellular pathways involved, which, for many of these cholinergic targets, appear to encompass both neuronal and non-neuronal elements, including the immune system.

Overall, these preclinical studies underscore the considerable potential of cholinergic compounds in the management of PNP, warranting the initiation of clinical trials.

## Data Availability

Data sharing is not applicable.

## Supplementary Materials

The following supporting information can be downloaded at: https://www.mdpi.com/article/10.3390/ph16101363/s1. Table S1. Description of animal models of peripheral neuropathic pain.
