# Supplementary material for: Exploring Cholinergic Compounds for Peripheral Neuropathic Pain Management: A Comprehensive Scoping Review of Rodent Model Studies
Source: Pharmaceuticals (Basel). 2023 Sep 27;16(10):1363. doi: 10.3390/ph16101363 (PMC10609809; doi:10.3390/ph16101363)
Supplement: Supplementary file 1 [file pharmaceuticals-16-01363-s001.zip › pharmaceuticals-2566372-supplementary.pdf]

Suplementary Material: Exploring Cholinergic Compounds for Peripheral Neuropathic Pain Management: A Comprehensive Scoping Review of Rodent Model Studies

Edouard Montigné and David Balayssac

Table S1. Description of animal models of peripheral neuropathic pain.

Neuropathic pain	Author and Year	Pain model	Ref
Traumatic	Liang et al., 2022	CCI	[91]
Traumatic	Bagdas et al., 2021	CCI	[74]
Traumatic	Wang et al., 2019	CCI	[81]
Traumatic	Bagdas et al., 2018	CCI	[45]
Traumatic	Li et al., 2018	CCI	[58]
Traumatic	Li et al., 2016	CCI	[104]
Traumatic	Bagdas et al., 2016	CCI	[72]
Traumatic	Luo et al., 2015	CCI	[87]
Traumatic	Papke et al., 2015	CCI	[77]
Traumatic	Bagdas et al., 2015	CCI	[79]
Traumatic	Di Cesare Mannelli et al., 2014a	CCI	[84]
Traumatic	Freitas et al., 2013	CCI	[69]
Traumatic	Loram et al., 2012	CCI	[76]
Traumatic	Marinelli et al., 2012	CCI	[39]
Traumatic	Holtman et al., 2011	CCI	[89]
Traumatic	Bagdas et al., 2011	CCI	[42]
Traumatic	Cheng et al., 2011	CCI	[62]
Traumatic	Pacini et al., 2010	CCI	[75]
Traumatic	Holtman et al., 2010	CCI	[54]
Traumatic	Vincler et al., 2006	CCI	[92]
Traumatic	Koga et al., 2019	PSL	[108]
Traumatic	Kiguchi et al., 2018	PSL	[56]
Traumatic	Saika et al., 2015	PSL	[53]
Traumatic	Gong et al., 2015	PSL	[68]
Traumatic	Kiguchi et al., 2012	PSL	[47]
Traumatic	Napier et al., 2012	PSL	[82]
Traumatic	Klimis et al., 2011	PSL	[83]
Traumatic	Ueda et al., 2011	PSL	[57]
Traumatic	Ueda et al., 2010	PSL	[63]
Traumatic	Song et al., 2008	PSL	[105]
Traumatic	Nevin et al., 2007	PSL	[85]
Traumatic	Takasu et al., 2006	PSL	[38]
Traumatic	Rashid and Ueda, 2002	PSL	[18]
Traumatic	Kesingland et al., 2000	PSL	[55]
Traumatic	Belgi et al., 2021	SNL	[100]
Traumatic	Ji et al., 2019	SNL	[71]
Traumatic	Brunori et al., 2018	SNL	[52]
Traumatic	Wood et al., 2017	SNL	[109]
Traumatic	Kimura et al., 2013	SNL	[20]
Traumatic	Kimura et al., 2012	SNL	[31]
Traumatic	Lee et al., 2011	SNL	[67]
Traumatic	Young et al., 2008	SNL	[48]
Traumatic	Ji et al., 2007	SNL	[64]
Traumatic	Josiah and Vincler, 2006	SNL	[50]

Traumatic	Rueter et al., 2003	SNL	[66]
Traumatic	Paqueron et al., 2001	SNL	[36]
Traumatic	Dhanasobhon et al., 2021	CM	[35]
Traumatic	Emril et al., 2016	SNCI	[41]
Traumatic	Ferrier et al., 2015	SNI	[32]
Traumatic	Wieskopf et al., 2015	SNI	[49]
Traumatic	Ortega-Legaspi et al., 2003	SNT	[107]
Traumatic	Abdin et al., 2006	TNT	[46]
Traumatic	Wang et al., 2017	CPNL	[106]
Traumatic	Zuo et al., 2015	CPNL	[34]
Traumatic	Liu et al., 2018	PSL + CCI	[80]
Traumatic	Xanthos et al., 2015	PSL + CCI	[51]
Traumatic	Satkunanathan et al., 2005	PSL + CCI	[101]
Traumatic	Nirogi et al., 2011	CCI + PSL + SNL	[65]
CIPN	Dyachenko et al., 2022	oxaPt	[93]
CIPN	Huynh et al., 2022	oxaPt	[97]
CIPN	Gajewiak et al., 2021	oxaPt	[99]
CIPN	Arias et al., 2020a	oxaPt	[73]
CIPN	Arias et al., 2020b	oxaPt	[90]
CIPN	Wang et al., 2019	oxaPt	[102]
CIPN	Romero et al., 2017	oxaPt	[95]
CIPN	Wood et al., 2017	oxaPt	[109]
CIPN	Christensen et al., 2017	oxaPt	[96]
CIPN	Pacini et al., 2016	oxaPt	[94]
CIPN	Yoon et al., 2015	oxaPt	[61]
CIPN	Ferrier et al., 2015	oxaPt	[32]
CIPN	Di Cesare Mannelli et al., 2014b	oxaPt	[70]
CIPN	Kanat et al., 2013	oxaPt	[43]
CIPN	Li et al., 2021	PTX	[103]
CIPN	Huynh et al., 2019	PTX	[98]
CIPN	Toma et al., 2019	PTX	[78]
CIPN	Kyte et al., 2018	PTX	[44]
CIPN	Favre-Guilmard et al., 2009	PTX	[40]
CIPN	Wala et al., 2012	VCT	[88]
CIPN	Nirogi et al., 2011	VCT	[65]
CIPN	Lynch et al., 2005	VCT	[59]
CIPN	Selvy et al., 2022	PTX + VCT + BTZ	[33]
Diabetic	Arias et al., 2020a	SZT	[73]
Diabetic	Calcutt et al., 2017	SZT	[110]
Diabetic	Nirogi et al., 2011	SZT	[65]
Diabetic	Chen et al., 2001	SZT	[37]
Diabetic	Saika et al., 2019	HFD	[60]

BTZ: bortezomib; CCI: chronic nerve injury; CIPN: chemotherapy-induced peripheral neuropathy; CM: cuff model; CPNL: common peroneal nerve ligation; HFD: high fat diet; oxaPt: oxaliplatin; PSL: partial sciatic nerve ligation; PTX: paclitaxel; SNCI: sciatic nerve crush injury; SNI: spared nerve injury; SNL: spinal nerve ligation; SNT: sciatic nerve transection; SZT: streptozotocin; TNT: tibial nerve transection; VCT: vincristine; nr: not reported.
